# Seasonal influenza vaccines differentially activate and modulate toll-like receptor expression within the tumor microenvironment

**DOI:** 10.3389/fonc.2024.1308651

**Published:** 2024-02-27

**Authors:** Kajal H. Gupta, Eileena F. Giurini, Andrew Zloza

**Affiliations:** ^1^ Division of Surgical Oncology, Department of Surgery, Rush University Medical Center, Chicago, IL, United States; ^2^ Division of Pediatric Surgery, Department of Surgery, Rush University Medical Center, Chicago, IL, United States; ^3^ Division of Surgical Oncology, Department of Surgery, The University of Texas Medical Branch, Galveston, TX, United States; ^4^ Division of Translational and Precision Medicine, Department of Internal Medicine, Rush University Medical Center, Chicago, IL, United States

**Keywords:** toll like receptor (TLR), innate immunity, flu vaccine, tumor microenvironment (TME), cancer vaccine

## Abstract

Toll-like receptors (TLRs) are well-known for their role in cancer development as well as in directing anti-tumor immunity. Because TLRs have also been implicated in the innate recognition of the influenza virus, it was of great interest to investigate the potential TLRs’ contribution to the reduction in tumor growth following intratumoral injection of an unadjuvanted influenza vaccine and the lack of antitumor response from an adjuvanted vaccine. In our previous publication, we showed that the unadjuvanted flu vaccine modulates TLR7 expression leading to anti-tumor response in a murine model of melanoma. Here, we show that the unadjuvanted and adjuvanted flu vaccines robustly stimulate different sets of TLRs, TLR3 and TLR7, and TLR4 and TLR9, respectively. In addition, the reduction in tumor growth and improved survival from intratumoral administration of the unadjuvanted vaccine was found to be diminished in TLR7-deficient mice. Finally, we observed that both vaccines have the capacity to modulate TLR expression on both innate and adaptive immune cells. Our findings add to the mechanistic understanding of the parameters that influence tumor outcomes in unadjuvanted and adjuvanted influenza vaccines.

## Introduction

The tumor immune microenvironment (TIME) encompasses innate immune factors that possess both pro-tumor and anti-tumor properties. Tumor progression, using the innate immune system, can be facilitated through two extremes: an immune-suppressed or chronically inflamed tumor microenvironment (TME) (1–3). A lack of immune infiltration in the TME is in part perpetuated through suppressive innate immune cell populations and the secretion of immunosuppressive cytokines, while the chronically inflamed TME is primarily mediated by sustained activation of pathogen recognition receptors (PRRs) and production of pro-inflammatory cytokines (2–4). Maintenance of these TME profiles culminates in tumor growth, angiogenesis, and invasion (2, 3). Though the extremes of immune activation within the TME are shown to promote tumorigenesis, an acute, potent inflammatory response induced within the TME can drive anti-cancer effects (5, 6). Activation of the innate immune system can convert suppressive tumor-associated macrophages (TAMs) to M1 macrophages and shape anti-tumor adaptive responses (7, 8).

Within the innate immune system are a set of PRRs that also possess a dual role in tumor outcomes, one such PRR is Toll-like receptors (TLRs). Upon recognition of pathogen-associated molecular patterns (PAMPs) by TLRs, dendritic cells (DCs) upregulate co-stimulatory molecules as well as major histocompatibility complex (MHC) I and II, leading to DC activation (9). Accordingly, TLR-activated DCs can induce anti-tumor T cell responses (10, 11). Additionally, TLR stimulation can shift suppressive immune cells to a pro-inflammatory or tumoricidal phenotype to initiate innate immune-mediated tumor eradication (12–14). However, TLR stimulation in an inappropriate context or with an inappropriate ligand can promote tumor progression. TLR-driven tumorigenesis primarily occurs through inducing tumor-promoting cytokine production and dysregulating TLR signaling (15, 16). Consequently, metastasis and resistance to apoptosis have been found to occur in a TLR activation-dependent manner (15–17).

In a previous study, we established that the seasonal influenza vaccine without adjuvants, as opposed to the adjuvanted vaccine, diminishes the growth of melanoma tumors. This is due to an increase in the CD8+ T cell population and a decrease in regulatory B cells in the TME (18). While prior research has shed light on the function of adaptive immunity in the intratumor effects of the two influenza vaccinations, the role of innate immune sensors remains unknown. In this study, we conduct a thorough analysis to understand the complex interplay of innate immune components in the context of unadjuvanted and adjuvanted influenza vaccinations, identifying a dualistic TLR profile associated with each formulation. Our research interests include determining the implications of these disparate TLR profiles in tumor outcome modification. We hope that by doing this systematic investigation, we will be able to provide a more comprehensive and nuanced understanding of the complex immunological landscape dictating the efficacy differences between unadjuvanted and adjuvanted influenza vaccinations in the setting of melanoma tumor dynamics.

## Methods

### Animals

B6 (C57BL/6J) and TLR7^-/^(B6.129S1-*Tlr7^tm1Flv^*/J) mice were purchased from The Jackson Laboratory at 6-10 weeks of age. Both male and female mice were used in this study. All animals were housed in specific-pathogen-free facilities and all experimental procedures were by policies approved by the Institutional Animal Care and Use Committee (IACUC) and Rush University Medical Center.

### Seasonal influenza vaccines & adjuvant

Two 2020-2021 FDA-approved seasonal influenza vaccines were purchased for these studies: Fluarix Quadrivalent (GlaxoSmithKline) and Fluad Quadrivalent (Seqirus). Addavax (Invivogen), a squalene-based oil in water adjuvant, was utilized to mimic adjuvant MF59 (Novartis). 50 µl of influenza vaccine or a PBS control were administered to mice via intratumoral injection. Removal of MF59 was completed using Amicon Ultra-0.5 centrifugal filter units with 30 kDa cutoff and a regenerated cellulose membrane. 500 µl of Fluad was added to the filter unit and washed with 250 µl acetone (3 times) followed by 250 µl PBS (3 times). The vaccine concentrate was subsequently collected and resuspended in 500 µl PBS.

### Cell culture

HEK-Blue TLR reporter cell lines (Invitrogen) transfected with plasmids containing a murine TLR and an NF-κB inducible secreted embryonic alkaline phosphatase (SEAP) reporter gene, were used to determine TLR stimulation. mTLR2, mTLR3, mTLR4, mTLR5, mTLR7, mTLR8, mTLR9, and mTLR13, and non-TLR expressing parental cell lines Null1, Null1k, Null1v, Null2, and Null2k (Invitrogen) were cultured using DMEM (Gibco), supplemented with 10% FBS (Corning), 100 units/mL penicillin (Gibco), 100 mg/mL streptomycin (Gibco), and 100 µg/mL normocin (Invivogen). Expression of TLR and SEAP plasmids were maintained with the addition of selective antibiotics blasticidin (10 µg/mL or 30 µg/mL, Invivogen), zeocin (100 µg/mL, Invivogen), or 1X HEK-Blue Selection (Invivogen). Murine melanoma cell line B16-F10 was cultured in DMEM supplemented with 10% FBS, 100 units/mL penicillin, 100 mg/mL streptomycin, and 100 µg/mL normocin.

### TLR activation assay

For evaluating TLR stimulation, 20 µl of each treatment was plated in triplicate in 96-well plates. TLR agonist positive controls were run simultaneously: TLR2 (Pam3CSK4, 1 μg/mL), TLR3 (Poly I: C, 1 μg/mL), TLR4 (LPS-EK, 100 ng/mL), TLR5 (FLA-ST, 2.5 μg/mL), TLR7 (CL264, 50 μg/mL), TLR8 (Poly(dT)/Imiquimod 10 μM), TLR9 (CpG ODN 2395, 5 μM), TLR13 (Sa19, 200 μg/mL). 1X PBS was used as a negative control. After plating the treatments, 50,000 mTLR or Null cells suspended in 180 µl HEK-Blue Detection (Invivogen) medium were added to the respective wells. All cells were incubated for 24 hours at 37°C and 5% CO_2_. Following incubation, TLR stimulation was quantified using a Cytation 3 plate reader (BioTek) measuring absorbance at 620_nm_.

### Tumor challenge

B6 and TLR7^-/^mice were anesthetized with isoflurane and challenged with 100,000 B16-F10 melanoma cells in 100 µl PBS via intradermal injection. Initiation of tumor treatment occurred when tumors ranged from 9-25 mm^2^ in size. Tumor development was monitored using Vernier calipers, where tumor area was determined by two measurements in perpendicular directions. To comply with IACUC policies, tumor-bearing mice were humanely sacrificed upon tumor measurements reaching 15 mm in either direction.

### Flow cytometry

Tumor-bearing mice were humanely sacrificed via carbon dioxide inhalation. For IL-10 staining, mice were administered (i.v., via retro-orbital injection) 50 µg of monensin (Sigma-Aldrich) dissolved in a diluted ethanol solution six hours before sacrifice. Tissues were processed as previously described (18). Extracellular staining for flow cytometry was performed with antibodies targeting CD3, CD11b, CD11c, CD20, CD45, F4/80, Ly6C, Ly-6G/Gr-1, MHCII, and TLR4. All antibodies were purchased from BioLegend, BD, eBioscience, or R&D Systems. An extracellular stain cocktail comprised of 1-5 µl/test in a total volume of 100 µl (made in PBS) was added to each sample and subsequently incubated in a dark environment at room temperature for 30 minutes. 0.25 µl/test of Live/Dead Fixable Aqua Dead Cell Stain Kit (405 nm excitation) (Invitrogen) was also added to the extracellular stain cocktail. Following extracellular staining, samples were washed twice with PBS before intracellular staining. For optimal intracellular staining, samples were permeabilized and fixed with 100 µl of Cytofix/Cytoperm (BD) for 15 minutes at 4°C. Samples were subsequently washed with 100 µl of 1X Perm/Wash Buffer (BD). Antibodies targeting IL-10, TLR3, TLR7, and TLR9 were used at 1-5 µl/test in a total volume of 100 µl (made in 1X Perm/Wash Buffer). Samples were incubated with intracellular stains for 30 minutes at 4°C, protected from light. Samples were subsequently washed with 200 µl of 1X Perm/Wash Buffer twice, followed by two 200 µl PBS washes. Flow cytometry was performed on the BD LSRFortessa. Flow cytometry analysis was completed using FlowJo (BD, version 10).

### Statistical analysis

Statistical analysis was performed using GraphPad Prism (version 9.1.2). For studies involving more than two groups, a 1-way ANOVA with Tukey correction was used to determine statistical significance. A two-way ANOVA was performed to determine statistical significance for studies with multiple time points. A Mantel-Cox log-rank test was used to determine the statistical significance of survival curves. For all studies, a p-value <0.05 was considered to be statistically significant.

## Results

### Unadjuvanted and adjuvanted seasonal influenza vaccines activate different sets of TLRs

**Figure 1:**
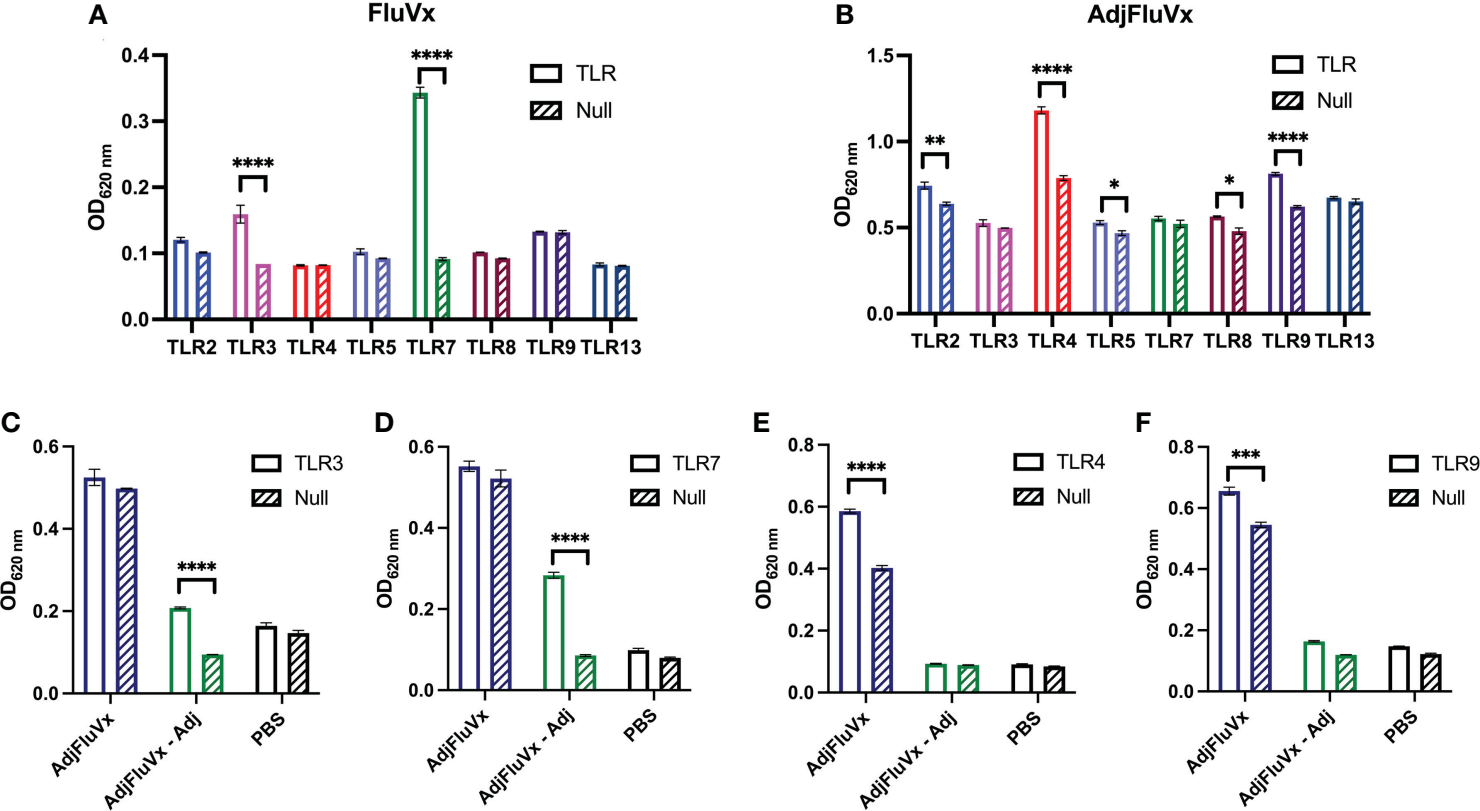
Unadjuvanted and adjuvanted seasonal influenza vaccines stimulate different sets of TLRs. **(A, B)** Activation of a murine TLR reporter panel and non-TLR expressing (Null) cells treated with an unadjuvanted (FluVx) or adjuvanted influenza vaccine (AdjFluVx). **(C, D)** Stimulation of TLRs previously not activated by AdjFluVx with an adjuvant-removed influenza vaccine. **(E, F)** Stimulation of TLRs previously activated by AdjFluVx with an adjuvant-removed influenza vaccine. Data are representative of three independent experiments run in triplicate. *P ≤ 0.05, **P ≤ 0.01, ***P ≤ 0.001, ****P ≤ 0.0001; two-way ANOVA with Sidak’s correction for multiple comparisons. Values represent mean ± S.E.M.

Host recognition of influenza virus infection begins with the detection of PAMPs by the innate immune system (19–21). Given that single-stranded RNA (ssRNA) from the influenza virus is a recognized PAMP, we sought to determine the TLR stimulatory potential of an unadjuvanted seasonal influenza vaccine (FluVx), as it contains inactivated influenza virus, and importantly, we have previously established its antitumor effects (18). Following FluVx treatment of a murine TLR reporter panel comprising cell lines for TLR2, TLR3, TLR4, TLR5, TLR7, TLR8, TLR9, and TLR13, both TLR3 and TLR7 were significantly stimulated compared to a non-TLR expressing Null cell line, (**Figure 1A**). Following these findings that FluVx activated several TLRs, the TLR stimulatory profile of an adjuvanted seasonal influenza vaccine (AdjFluVx) was evaluated. AdjFluVx was found to stimulate TLR2, TLR4, TLR5, TLR8, and TLR9, but most notably TLR4 and TLR9 (**Figure 1B**) compared to Null cell lines. To further elucidate the TLR-modulatory effects of the addition or removal of a squalene-based MF-59-like adjuvant (Adj) to the vaccines, TLR activation by Adj was evaluated. Adj was found to activate many of the same TLRs as AdjFluVx, and similarly most robustly stimulating TLR4 and TLR9, (**Supplementary Figure S1**) thus confirming that the TLR stimulatory profile observed with AdjFluVx is driven by its inclusion of Adj. To further confirm these findings, we sought to identify a baseline activation status of the cells. Both TLR and Null cell lines were treated with a PBS control and demonstrated no significant changes in activation between the TLR and Null cell lines (**Supplementary Figure S2**). After confirming our findings that FluVx and AdjFluVx stimulate different sets of TLRs, the next step was to ascertain any difference in TLR activation upon removal of Adj (MF59) from AdjFluVx. Treatment of TLR3 and TLR7 cell lines with AdjFluVx was consistent with prior experiments, no significant TLR stimulation was observed. However, when Adj was removed from AdjFluVx, significant TLR3 and TLR7 stimulation was uncovered, more akin to that of FluVx (**Figures 1C, D**).

Concurrently, the removal of the adjuvant from AdjFluVx yielded loss in its ability to activate TLR4 and TLR9 (**Figures 1E, F**). As expected, addition of Adj to FluVx diminished its ability to stimulate TLR3 and TLR7, while increasing TLR4 and TLR9 stimulation (**Supplementary Figure S2**). Altogether, these data indicate that FluVx and AdjFluVx stimulate discrete sets of TLRs, determined by the absence or presence of Adj.

### TLR7 contributes to improved tumor outcomes from the intratumoral unadjuvanted seasonal influenza vaccine

**Figure 2:**
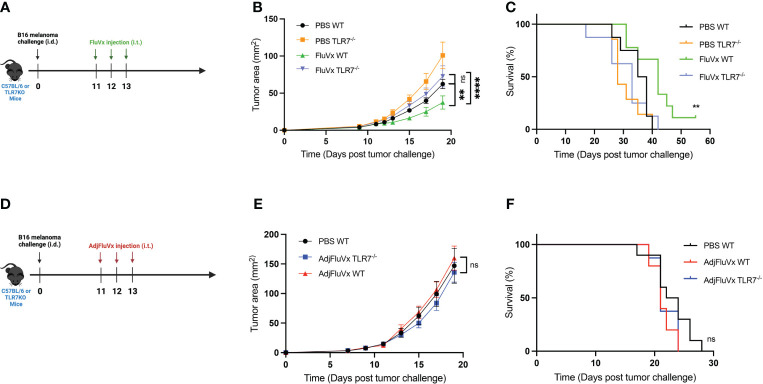
Reduction in tumor growth and improved survival from intratumoral administration of seasonal influenza vaccine is mediated by TLR7. **(A)** Experimental design. n = 8-10 mice per group. **(B)** Tumor growth curves of experiment described in **(A). (C)** Survival curves from experiment described in **(A). (D)** Experimental design. n = 10 mice per group. **(E)** Tumor growth curves of experiment described in **(D)** n = 10 mice per group. **(F)** Survival curves from experiment described in **(D)** Data are representative of at least two independent experiments. ns, not significant, **P < 0.01, **** *P* ≤ 0.0001; 2-way ANOVA with Tukey correction, Mantel-Cox log-rank test. Values represent mean ± S.E.M.

To determine any association between *in vitro* TLR activation and the impact of TLRs in the antitumor effects of FluVx, we conducted an experiment with TLR7-deficient mice. TLR7-deficient (TLR7^-/-^) mice bearing B16 melanoma tumors treated with FluVx did not experience reduction in tumor progression which was found in TLR7-competent, C57BL/6J (WT) mice (**Figures 2A, B**). This finding suggests that the observed *in vitro* activation of TLR7 from FluVx is also important in facilitating its antitumor immunity. To further examine the role of TLR7 in tumor outcomes from FluVx treatment, we observed survival of FluVx-treated WT and TLR7^-/-^ mice. Not only was reduction in tumor growth abrogated in TLR7^-/-^ mice after FluVx treatment, but the prolonged survival was also diminished (**Figure 2C**). In accordance with our *in vitro* findings that AdjFluVx did not stimulate TLR7, intratumoral administration of AdjFluVx in WT and TLR7^-/-^ mice bearing B16 melanoma tumors did not experience any change in tumor progression (**Figures 2D, E**). Accordingly, no significant change in survival was observed between WT and TLR7^-/-^ treated with AdjFluVx (**Figure 2F**). These data indicate that TLR7 is an important mediator in the antitumor immune response from FluVx.

### Seasonal influenza vaccines modulate TLR expression on innate and adaptive immune cells within the tumor

**Figure 3:**
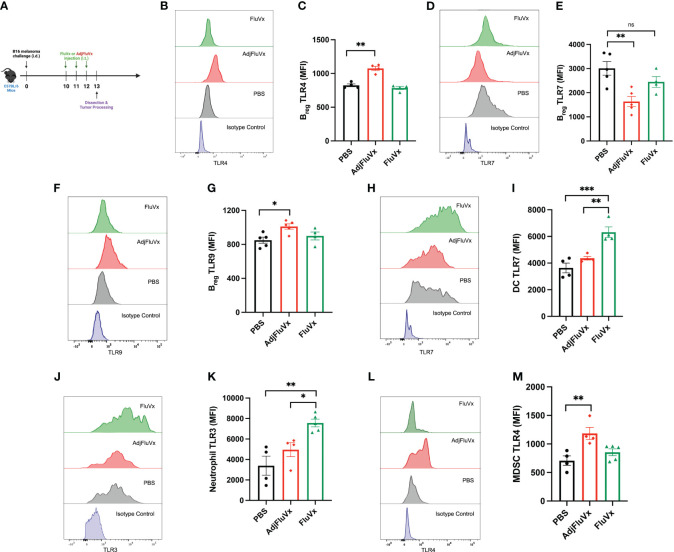
Intratumoral injection of unadjuvanted and adjuvanted seasonal influenza vaccines modulates TLR expression within the tumor microenvironment. TLR expression was measured by mean fluorescence intensity (MFI). **(A)** Experimental design. n = 3-5 tumors per group. **(B)** Representative flow cytometry histogram of TLR4 expression on regulatory B cells (B_regs_ (CD45+ CD20+ IL-10+) each treatment group. **(C)** TLR4 expression from intratumoral B_regs_ following experimental conditions described in **(A)**. **(D)** Representative flow cytometry histogram of TLR7 expression on B_regs_ from each treatment group. **(E)** TLR7 expression on intratumoral B_regs_ following experimental conditions described in **(A)**. **(F)** Representative flow cytometry histogram of TLR9 expression on B_regs_ from each treatment group. **(G)** TLR9 expression on intratumoral B_regs_ following experimental conditions described in **(A). (H)** Representative flow cytometry histogram of TLR7 expression on dendritic cells(DCs), DCs were defined as (CD45+ CD11c+ MHCII+). **(I)** TLR7 expression on intratumoral DCs following experimental conditions described in **(A)**. **(J)** Representative flow cytometry histogram of TLR3 expression on neutrophils, neutrophils were defined (CD45+ CD11b+ Ly6G/Gr-1+ F4/80-). **(K)** TLR3 expression on intratumoral neutrophils following experimental conditions described in **(A)**. **(L)** Representative flow cytometry histogram of TLR4 on myeloid-derived suppressor cells (MDSCs), MDSCs were defined as (CD45+ CD11b+ Ly6C+ Ly6G/Gr-1- F4/80+). **(M)** TLR4 expression on intratumoral MDSCs following experimental conditions described in **(A)**. ns, not significant, *P < 0.05, **P < 0.01, ***P < 0.001; 1-way ANOVA with Tukey’s multiple comparisons test. Values represent mean ± S.E.M.

In several contexts, TLR expression is upregulated in the presence of its respective stimulating ligand (22–26). Accordingly, we assessed the modulatory capacity of AdjFluVx and FluVx on TLR expression within the tumor by flow cytometry (**Figure 3A**), gating strategy summarized in **Supplementary Figure S3**. Changes in TLR expression on regulatory B cells (B_regs_) was of particular interest, as TLR expression on B_regs_ has been previously recognized (27–29). After AdjFluVx treatment, intratumoral B_regs_ were found to have significantly upregulated both TLR4 (**Figures 3B, C**). Unexpectedly, B_regs_ from AdjFluVx treatment also decreased TLR7 expression on intratumoral B_regs_ (**Figures 3D, E**), suggesting AdjFluVx has the ability to upregulate the TLRs it stimulates that were observed *in vitro*. Similarly, TLR9 expression on B_regs_ was increased in the AdjFluVx treated groups compared to PBS and FluVx treated groups (**Figures 3F, G**). In addition, TLR expression profiling was conducted for dendritic cells (DCs). FluVx treated tumors were found to significantly upregulate TLR7 on DCs and TLR3 on neutrophils (**Figures 3H–K**), suggesting that FluVx is also capable of modulating the expression of the TLRs it activates. Finally, intratumoral administration of AdjFluVx was found to significantly upregulate TLR4 expression on tumor localized myeloid derived suppressor cells (MDSCs) (**Figures 3L, M**). These data indicated that both FluVx and AdjFluVx can modulate the TLRs expressed on immune cells that are encompassed within the tumor.

## Discussion

TLR activation emerges as a critical determinant in the complex landscape of the TME, with the dualistic ability to either promote or restrict tumor progression. Given the disparities in responses reported after intratumoral delivery of FluVx and AdjFluVx, as well as influenza’s inherent TLR activation potential, research into the *in vivo* influence of TLRs on these effects became critical.

Our research found that FluVx and AdjFluVx had distinct TLR activation profiles, with no overlap in TLR activation between the two vaccine formulations. TLR4 and TLR9 were shown to be the most responsive to the adjuvanted vaccine, AdjFluVx. Previous research has linked TLR4 and TLR9 activation to cancer growth in a variety of situations (30–32). As a result, our previously documented maintenance of tumor development following AdjFluVx treatment could be attributed to TLR4 and TLR9 activation, supporting an immunosuppressed tumor microenvironment.

FluVx, on the other hand, was found to activate TLR3 and TLR7. These TLRs have gotten a lot of interest because of their therapeutic potential in cancer (33, 34). Notably, the TLR7 agonist imiquimod is used to treat basal cell carcinoma and encouraging results have been observed by simultaneously targeting TLR3 and TLR7. TLR3 and TLR7 identification of viral-based PAMPs inside the tumor microenvironment is thought to initiate antitumor immunity following intratumoral FluVx therapy.

Following the discovery that seasonal influenza vaccinations activate TLRs *in vitro*, we focused on establishing TLR7’s role in B16 melanoma tumor development after therapy. Using a TLR7-deficient mouse model, we observed that TLR7 deficiency promotes tumor growth and decreases survival rates. However, intratumoral FluVx treatment reduced tumor development and increased survival rates. This attenuation is most likely owing to a lack of downstream signaling caused by the interaction of FluVx and TLR7.

Concurrently, the lack of observable effects on tumor progression following intratumoral injection of AdjFluVx in TLR7-deficient animals highlights the insignificant role of TLR7 activation in influenza vaccines containing a squalene oil-in-water adjuvant.

FluVx and AdjFluVx were found to affect the expression of certain TLRs inside the tumor microenvironment, supporting the idea that TLR stimulatory ligands can upregulate target TLRs. The elevation of TLR4 and TLR9 expression on regulatory B cells following AdjFluVx therapy was particularly notable, matching with previous findings that TLR4 and TLR9 activation stimulates the release of immunosuppressive cytokines such as IL-10 from immune cells, including regulatory B cells (28). Consistent with our previous findings that IL-10 blockade allows the adjuvanted influenza vaccine to suppress tumor growth, these findings suggest that AdjFluVx treatment upregulates and activates TLR4 and TLR9 on regulatory B cells in a feed-forward manner, likely contributing to sustained IL-10 production and impeding any antitumor response (35).

TLR7 expression on regulatory B cells within the tumor was also reduced following AdjFluVx therapy. TLR7 activation, despite being expressed on regulatory B cells, has been identified as a negative regulator for these cells (1), decreasing IL-10 production and reducing splenic regulatory B cell numbers (29). TLR7 downregulation by AdjFluVx may contribute to the maintenance of regulatory B cell populations and IL-10 production within the tumor, encouraging tumor growth (30).

Furthermore, the elevation of TLR7 expression on tumor dendritic cells following FluVx therapy is significant. Given that FluVx treatment increases dendritic cell populations within the tumor (18), this finding suggests a possible mechanism in which FluVx upregulates and activates TLR7 signaling on dendritic cells, subsequently activating downstream targets such as NF-kB or MAPK/ERK pathways that regulate cell proliferation. TLR7 signaling may also activate dendritic cells, boosting tumor antigen presentation and promoting tumor-targeting CD8+ T cell responses. TLRs are expressed on both immune and tumor cells in the TME and play a dual role, triggering both anti-tumor (innate and adaptive immunity) and pro-tumor (cell proliferation, migration, invasion, and cancer stem cell maintenance), and have been linked to a variety of cancers including glioblastoma (31), breast cancer, melanoma, and brain tumors (32). TLR7 has been targeted as a potential therapeutic for hepatocellular carcinoma (33).

In conclusion, our data highlight the multifaceted and dualistic role of TLRs in cancer. Intratumoral FluVx therapy reveals the anticancer potential of TLR signaling, with TLR7 identified as a factor to tumor progression reduction. TLRs activated by AdjFluVx, on the other hand, increase the generation of immunosuppressive cytokines, sustaining an environment favorable to tumor growth. The minimal adverse effects and strong safety profile of FluVx established by the FDA (34) in combination with these findings giving light to intriguing immunotherapeutic targets, position FluVx as a promising and well-tolerated cancer immunotherapy.

## Data availability statement

The original contributions presented in the study are included in the article/**Supplementary Material**. Further inquiries can be directed to the corresponding authors.

## Ethics statement

All the experiments were conducted in accordance with procedures approved by the InstitutionalAnimal Care and Use Committee (IACUC) and Institutional Biosafety Committee at Rush University Medical Center.

## Author contributions

KG: Formal analysis, Investigation, Methodology, Supervision, Visualization, Writing – original draft, Writing – review & editing. EG: Formal analysis, Investigation, Methodology, Writing – review & editing. AZ: Funding acquisition, Resources, Writing – review & editing.

## Supplementary material

Supplementary Figure 1 Squalene-based adjuvant found in adjuvanted influenza vaccine stimulates TLR2, TLR4, TLR5, TLR8, and TLR9. **(A)** Activation of a murine TLR reporter panel and parental, non-TLR transfected Null cell lines with squalene oil-in-water adjuvant (Adj). **(B)** Detection of baseline activation of murine TLR reporter panel and Null cell lines with PBS control. Data are representative of three independent experiments run in triplicate. **P* < 0.05, ***P* < 0.01, ****P* < 0.001, *****P* < 0.001; two-way ANOVA with Sidak’s correction for multiple comparisons. Values represent mean ± S.E.M.

Supplementary Figure 2 The addition of squalene oil-in-water adjuvant to unadjuvanted seasonal influenza vaccine diminishes intrinsic TLR stimulation and promotes activation of other TLRs. Ratio of TLR reporter cell stimulation to parental Null cell stimulation when treated with unadjuvanted influenza vaccine (FluVx) or adjuvant (Adj) added to unadjuvanted influenza vaccine (FluVx + Adj). Data are representative of three independent experiments run in triplicate.

Supplementary Figure 3 Flow cytometry gating strategy of intratumoral immune cell populations. **(A)** Flow cytometry gating of regulatory B cells (Bregs). Bregs were defined as CD45+ CD20+ CD3- IL-10+. **(B)** Flow cytometry gating of dendritic cells (DCs). DCs were defined as CD45+ CD11c+ MHCII+. **(C)** Flow cytometry gating of myeloid derived suppressor cells (MDSCs). MDSCs were defined as CD45+ CD11b+ Ly6C+ Ly6G-. **(D)** Flow cytometry gating of neutrophils. Neutrophils were defined as CD45+ CD11b+ Ly6G+ F4/80-. **(E)** CD45 isotype control. **(F)** CD45+ cells stained with CD20 or CD3 isotype control. **(G)** CD45+ cells stained with CD3 or CD20 isotype control. **(H)** CD45+ CD20+ cells stained with IL-10 isotype control. **(I)** CD45+ MHCII+ cells stained with CD11c isotype control. **(J)** CD45+ cells stained with MHCII isotype control. (K) CD45+ cells stained with F4/80 isotype control. **(L)** CD45+ cells stained with Ly6G isotype control. **(M)** CD45+ cells stained with CD11b isotype control. **(N)** CD45+ cells stained with Ly6C isotype control.
