# Supplementary figures and images for: Seasonal influenza vaccines differentially activate and modulate toll-like receptor expression within the tumor microenvironment
Source: Front Oncol. 2024 Feb 27;14:1308651. doi: 10.3389/fonc.2024.1308651 (PMC10928891; doi:10.3389/fonc.2024.1308651)

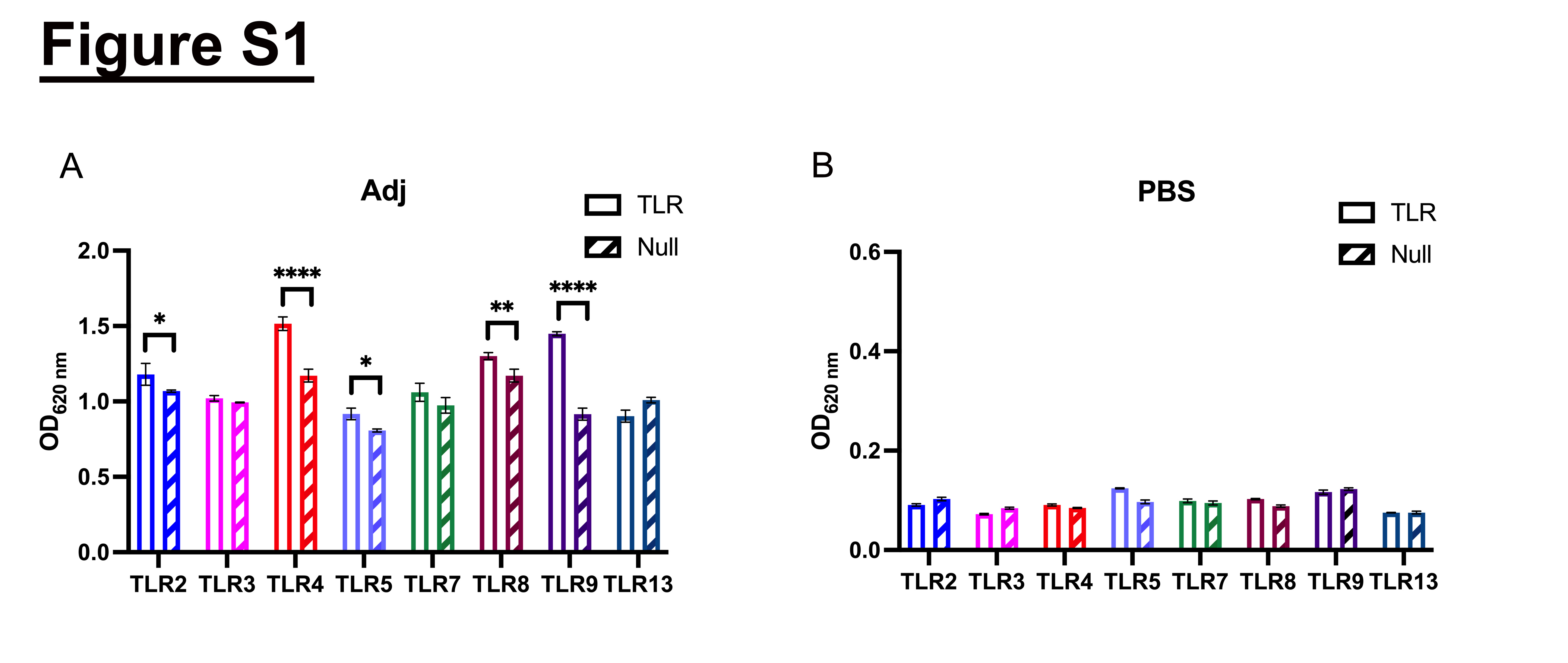

Supplement: Supplementary Figure 1 — Squalene-based adjuvant found in adjuvanted influenza vaccine stimulates TLR2, TLR4, TLR5, TLR8, and TLR9. (A) Activation of a murine TLR reporter panel and parental, non-TLR transfected Null cell lines with squalene oil-in-water adjuvant (Adj). (B) Detection of baseline activation of murine TLR reporter panel and Null cell lines with PBS control. Data are representative of three independent experiments run in triplicate. *P < 0.05, **P < 0.01, ***P < 0.001, ****P < 0.001; two-way ANOVA with Sidak’s correction for multiple comparisons. Values represent mean ± S.E.M. [file Image_1.jpeg]

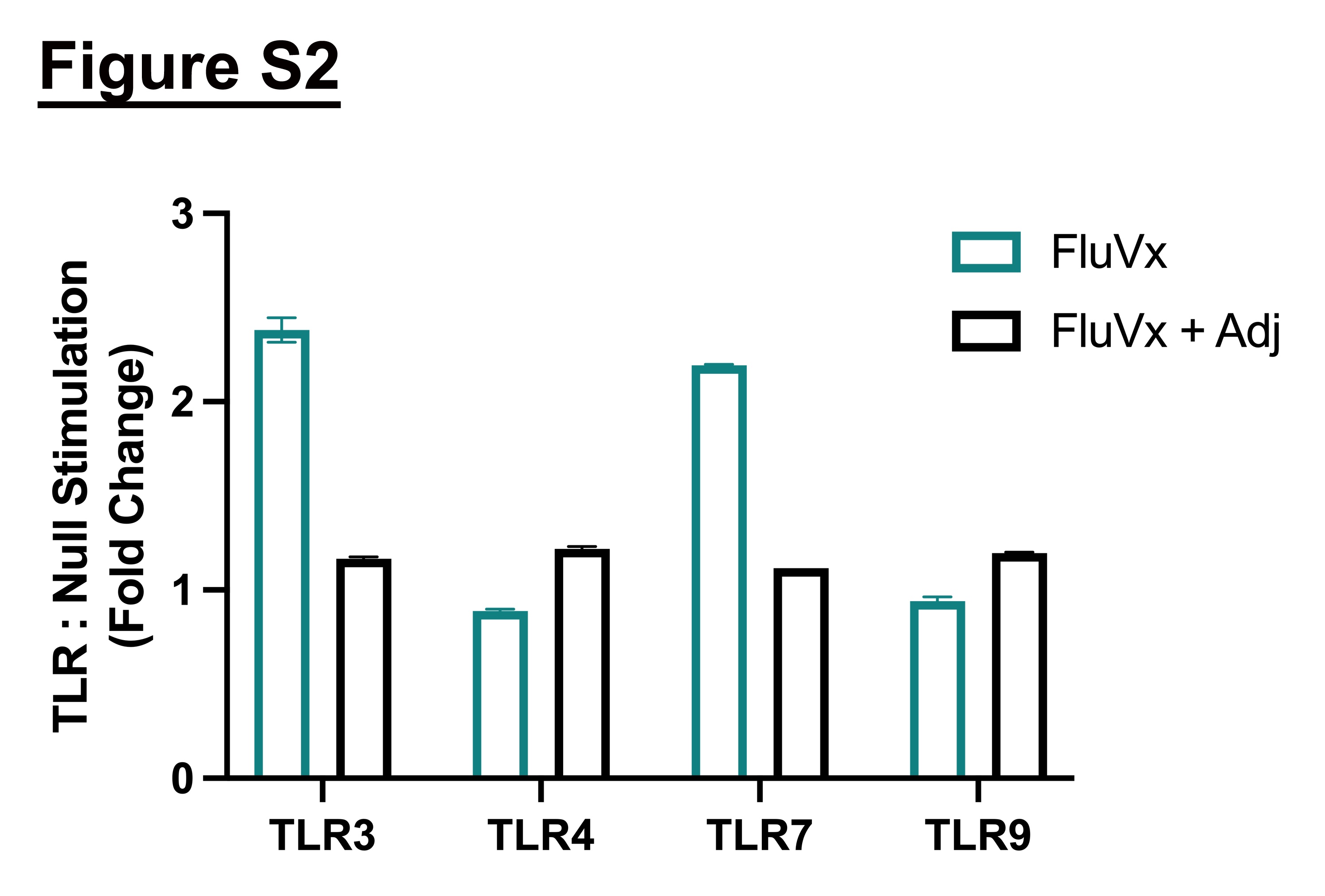

Supplement: Supplementary Figure 2 — The addition of squalene oil-in-water adjuvant to unadjuvanted seasonal influenza vaccine diminishes intrinsic TLR stimulation and promotes activation of other TLRs. Ratio of TLR reporter cell stimulation to parental Null cell stimulation when treated with unadjuvanted influenza vaccine (FluVx) or adjuvant (Adj) added to unadjuvanted influenza vaccine (FluVx + Adj). Data are representative of three independent experiments run in triplicate. [file Image_2.jpeg]

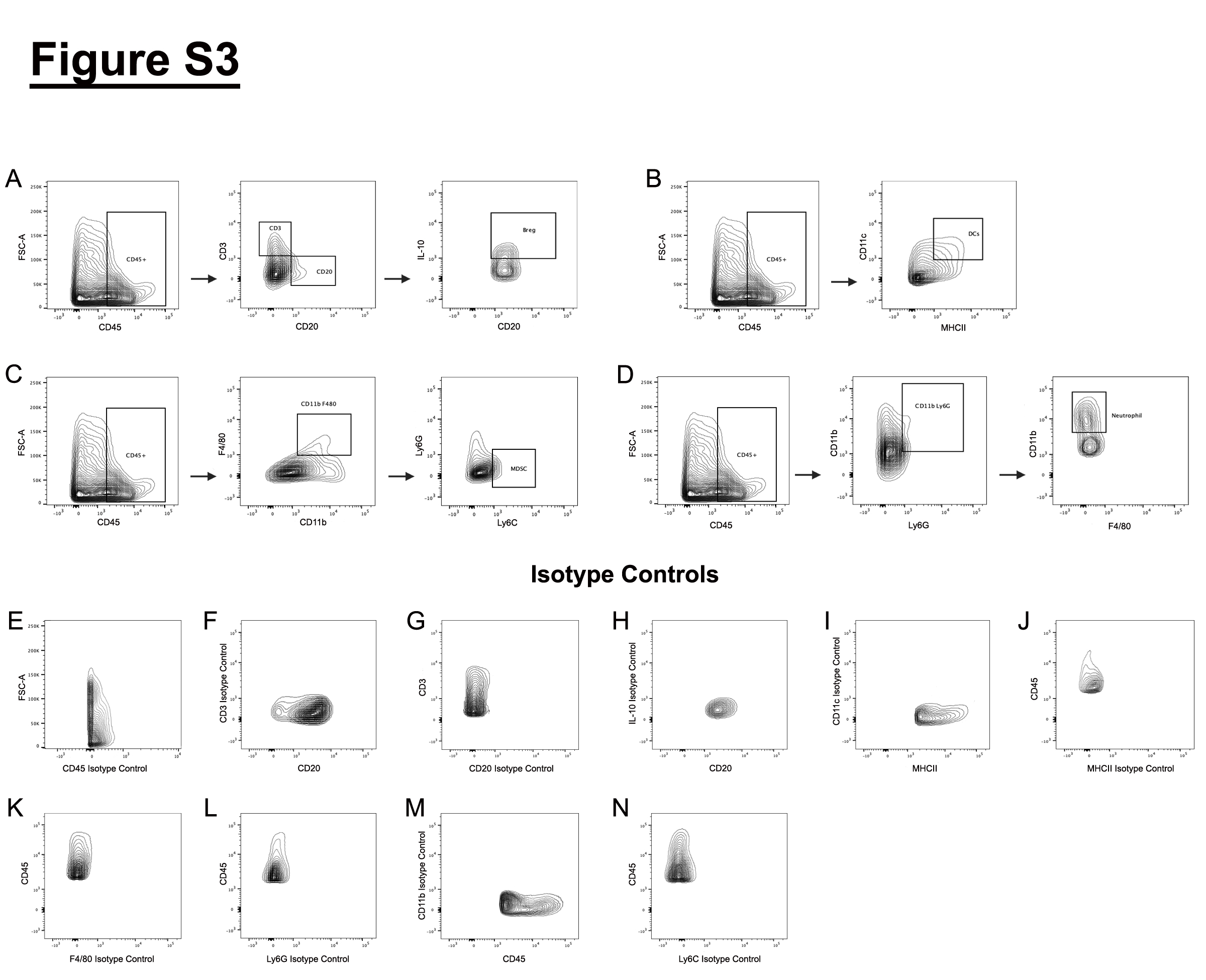

Supplement: Supplementary Figure 3 — Flow cytometry gating strategy of intratumoral immune cell populations. (A) Flow cytometry gating of regulatory B cells (Bregs). Bregs were defined as CD45+ CD20+ CD3- IL-10+. (B) Flow cytometry gating of dendritic cells (DCs). DCs were defined as CD45+ CD11c+ MHCII+. (C) Flow cytometry gating of myeloid derived suppressor cells (MDSCs). MDSCs were defined as CD45+ CD11b+ Ly6C+ Ly6G-. (D) Flow cytometry gating of neutrophils. Neutrophils were defined as CD45+ CD11b+ Ly6G+ F4/80-. (E) CD45 isotype control. (F) CD45+ cells stained with CD20 or CD3 isotype control. (G) CD45+ cells stained with CD3 or CD20 isotype control. (H) CD45+ CD20+ cells stained with IL-10 isotype control. (I) CD45+ MHCII+ cells stained with CD11c isotype control. (J) CD45+ cells stained with MHCII isotype control. (K) CD45+ cells stained with F4/80 isotype control. (L) CD45+ cells stained with Ly6G isotype control. (M) CD45+ cells stained with CD11b isotype control. (N) CD45+ cells stained with Ly6C isotype control. [file Image_3.jpeg]
